# Exfoliation of the Sockets of Two Teeth

**Published:** 1858-01

**Authors:** 


*Exfoliation of the Sockets of two Teeth.*—Dr. R. L. Jones, dentist, of Columbia, Ky., sent to the senior editor, the sockets of the right superior central and lateral incisors which he removed from the mouth of a man about ten months ago. The alveoli were in a necrosed condition—the dead bone extending to the cavity of the nose, so that, for some time after its removal water taken into the mouth escaped from the nose. The inflammation which caused the death of the bone is supposed to have been caused by a cold contracted some weeks before.

